# Micro-Technologies for Assessing Microbial Dynamics in Controlled Environments

**DOI:** 10.3389/fmicb.2021.745835

**Published:** 2022-01-28

**Authors:** Shanna-Leigh Davidson, Tagbo H. R. Niepa

**Affiliations:** ^1^Department of Chemical and Petroleum Engineering, University of Pittsburgh, Pittsburgh, PA, United States; ^2^Department of Bioengineering, University of Pittsburgh, Pittsburgh, PA, United States; ^3^Department of Civil and Environmental Engineering, University of Pittsburgh, Pittsburgh, PA, United States; ^4^Department of Mechanical Engineering and Materials Science, University of Pittsburgh, Pittsburgh, PA, United States; ^5^Center for Medicine and the Microbiome, University of Pittsburgh, Pittsburgh, PA, United States; ^6^The McGowan Institute for Regenerative Medicine, University of Pittsburgh, Pittsburgh, PA, United States

**Keywords:** microfluidics, nanocultures, microarrays, unculturable microbes, micromachined devices

## Abstract

With recent advances in microfabrication technologies, the miniaturization of traditional culturing techniques has provided ideal methods for interrogating microbial communities in a confined and finely controlled environment. Micro-technologies offer high-throughput screening and analysis, reduced experimental time and resources, and have low footprint. More importantly, they provide access to culturing microbes *in situ* in their natural environments and similarly, offer optical access to real-time dynamics under a microscope. Utilizing micro-technologies for the discovery, isolation and cultivation of “unculturable” species will propel many fields forward; drug discovery, point-of-care diagnostics, and fundamental studies in microbial community behaviors rely on the exploration of novel metabolic pathways. However, micro-technologies are still largely proof-of-concept, and scalability and commercialization of micro-technologies will require increased accessibility to expensive equipment and resources, as well as simpler designs for usability. Here, we discuss three different miniaturized culturing practices; including microarrays, micromachined devices, and microfluidics; advancements to the field, and perceived challenges.

## Introduction

From pharmaceuticals and food preservation to renewable energies, such as biofuels, the metabolic products harnessed from microbial organisms are reliant on culture-dependent isolation, purification and scale-up. Although there are thousands of species we now know to exist through culture-independent methods, the fact remains that majority of these species are unable to be cultivated using traditional culturing methods, and are aptly named, “Microbial Dark Matter” (Lok, 2015; Dance, 2020). In fact, since Koch developed standardized isolation and maintenance protocols for the domestication of microorganisms in the late 1800s, cultivation techniques have remained much the same (Srinivasan et al., 2015). Although imperative for the controlled study of monocultures, traditional culturing techniques leave much to be desired for the full cultivation of complex microbiomes, whether they are environmental, such as soil or marine, or animal and human derived. These microbiomes are diverse, constituent not only of microbes that we commonly use today, but also of species that live in low abundance, are recalcitrant and fastidious, as well as “viable but non-culturable” (VBNC) organisms; a term introduced by Xu et al. (1982) to describe the set of adaptive strategies taken on by microorganisms to persist in adverse conditions for long periods of time (Ramamurthy et al., 2014; Pienaar et al., 2016; Göing and Jung, 2021). The inability to culture these microorganisms in a laboratory setting presents a non-trivial, but not impossible, challenge (Bodor et al., 2020). However, the advancement of intra- and inter-cellular microbial dynamics and discovery of novel metabolic products is imperative to the cultivation of these species.

It is not since the last 30–40 years that researchers have really begun to appreciate the expansive heterogeneity of the micro-biosphere. One of the main drivers for the discovery and cultivation of microbial dark matter has been the persistently growing problem of antibiotic drug resistant organisms (ADROs), a global issue that presently costs the US healthcare system an estimated $20 billion annually in direct costs, with a further $35 billion estimated in lost productivity (Zhen et al., 2019; CDC, 2020). Attempting to find novel drug targets, researchers have overmined and exhausted any secondary metabolites available from the small cohort of microbial species that can currently be isolated and cultivated in the lab. However, it is also hypothesized that for the 10^11^−10^12^ microbial species that are estimated to inhabit the Earth, more than 99% have yet to be discovered and an even smaller fraction are able to be cultured by current techniques (Locey and Lennon, 2016). The push to discover new microbial species has spurred an interest in designing innovative, novel culturing techniques that will lead to the cultivation of species never seen before (Whitesides, 2006).

A major challenge in cultivating unculturable microorganisms has been the distinct lack of knowledge and understanding in mimicking the optimal local environment for these species (Stewart, 2012; Chaudhary et al., 2019). While modifications to standard laboratory growth media can be simple manipulations, such as adjusting temperature, pH, osmotic pressure, and aerobic conditions, we now also understand that other biotic factors may be necessary too. For example, one factor that might play into the growth of “unculturable” species as well as VBNCs, includes synergistic interactions amongst species, whereby growth of one might be dependent on the production of secondary metabolites of another (Park et al., 2011; Bodor et al., 2020). These biotic factors are impossible to simulate with traditional cultivation methods. Moreover, traditional culturing techniques are low throughput, resulting in tedious experimental methods, significant lag time between start of experiment and data analysis, and extraneous use of resources (Zengler et al., 2002). This has resulted in a single sentiment within the research community: How can we exploit the natural microbiome environment, whilst attaining ultra- high throughput efficiency and parallel sampling within a controlled lab setting?

**FIGURE 1:**
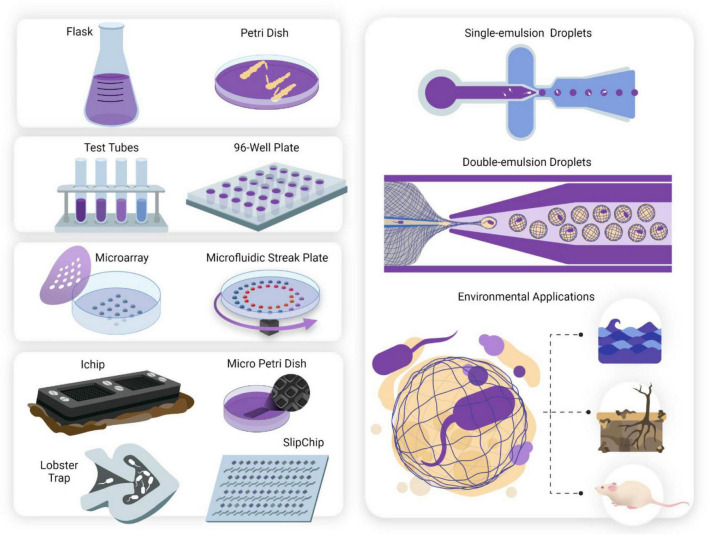
The miniaturization of cell culturing techniques has revolutionized single-cell studies for culturing microorganisms deemed “unculturable.” Conventional culturing methods (mL—L). Anaerobic jars, flasks and Petri dishes have been used since they were first developed in the 1800s and still form the basis for traditional cell culturing today. **Serial dilutions and microwell plates** (μL—mL). These mark the first scaling down of culturing techniques and allow for quantification of cell growth and automated screening**. Microarray technologies** (nL—μL). Microarray printing of droplets allows for highly automated single-cell analyses and investigation of metabolic enzymatic reactions. Two examples include the Microbe Observation and Cultivation Array (MOCA) (Gao et al., 2013) and the Microfluidic Streak Plate (MSP) (Jiang et al., 2016). **Micromachined devices** (nL—μL). Micromachined devices make use of microfabrication techniques to design devices containing microwells or chambers for cultivating and screening microbes. Examples of such devices include the ichip (Nichols et al., 2010) micro-Petri dish (Ingham et al., 2007), lobster traps (Connell et al., 2010), and SlipChip (Ma et al., 2014). **Microfluidic technologies** (pL—nL). Microfluidics offer observations with flow dynamics in microfluidic channels, and isolation and compartmentalization of cells in the form of single (Chang et al., 2015) and double (Niepa et al., 2016) emulsion droplets. Droplets can be aqueous or gel phase (Lin et al., 2011), offering tunable constraints for study design. Culturing the “unculturable”- from macroscale to nanoscale. Untapped potential lies awaiting exploration in microbiomes such as marine, animal and soil systems. Discoveries of novel species may influence drug discovery, human health and other fields including bioremediation and agriculture.

The answer to this lies in the miniaturization of culturing techniques (Figure 1; Weibel et al., 2007). With the development of microfabrication, there has been an influx of innovatively designed microdevices targeted toward achieving ultra-low sample volumes; hence, interrogating the immediate microenvironment of microorganisms (Weibel and Whitesides, 2006; Hansen et al., 2016). The aspect of the microenvironment becomes a significant design requirement for the cultivation of “unculturable” species, notably for necessary biotic factors (secondary synergistic/antagonistic metabolite interactions between species, quorum sensing, etc.) that must be controlled in a simulated environment (Franklin et al., 2015). Microdevices offer ultra-high throughput and parallel sampling, which results in reduced experimental times, smaller volume of sample and reagents required, and overall, less expensive experimental design. Using such microdevices, natural microniches can now be brought into the lab, or simulated, to tease apart the intricate intra- and inter-species relationships that make up the rich biodiversity that allow our natural world to flourish (Chaudhary et al., 2019).

Understanding novel metabolic pathways arising from members of various microbiomes will impact all facets of life. Just a few applications will include drug discovery in the times of antibiotic resistance, better diagnostics in point-of-care (POC) healthcare, bioremediation of hydrocarbons and plastics, and even smart probiotics that inhibit colonization of opportunistic pathogens (Weibel et al., 2007). Here, microdevice technologies are discussed in three broader categories: microarrays, micromachined devices, and microfluidics. We also highlight the benefits and drawbacks of these methods, summarized in Table 1.

**TABLE 1:** A table of comparison of advantages and disadvantages of discussed micro-technologies.

	Culture Flask	Agar Petri plate	Microwell plate	Microarray cell printing	Micromachined devices	Microfluidics	
						Single-emulsion	Double-emulsion
Culture volume	mL—L	mL	μL—mL	nL—μL	pL—μL	pL—nL	pL—nL
High throughput assay	−	−	+	++	+++	+++	+++
Parallel sampling	−	−	+	++	++	+++	+++
Reagent cost	+++	+++	++	+	+	+	+
Cell isolation and sorting	−	+	−	+	+++	++	+++
*In situ* cultivation	−	−	−	−	+++	−	+++
Cultivation period	1–7 days	∼ 2 weeks	48 h	24 h	Weeks—Months	Days	Days—Months
Imaging requirement	−	−	Confocal imaging reader	Light microscope	Light microscope	Light microscope	Light microscope
High-throughput data processing	−	−	+++	+++	+	++	++
Materials design and functionality	−	−	−	−	+++	−	+++
Manufacturing costs	−	−	−	+	++	+++	++

## Advances in Micro-Culturing Technology

The first success in miniaturizing sample volume in the lab came in the form of microwell plates, invented by Hungarian Dr. Gyula Takatsy in 1951. His invention revolutionized the way that titrations and serial dilutions were performed in the lab (Figure 1; Banks, 2009). However, microwell plates pose some disadvantages: they are not truly high-throughput and maintaining culture volume for an extended period proves problematic due to evaporation. Furthermore, accumulation of waste products within wells and limitations in oxygen transfer limit confluent cell growth. Lastly, microwell plates have low image resolution, making it difficult to visualize qualitative data (Ingham et al., 2007).

It has taken another 50 years to further develop the space of “micro-culturing” to achieve the standards required for high-throughput screening, but also in understanding how microorganisms interact with each other in complex ways. In miniaturizing culture conditions, one can isolate and compartmentalize microorganisms such that inter-species competition for space and resources is negated (Niepa et al., 2016). Such competition can often be seen with fast-growing species that mask slow growers. However, slow growing species make up a large proportion of microbial diversity, albeit in low abundance (Jiang et al., 2016). Therefore, there is huge untapped potential to explore in these spaces and the micro-technologies described here provide the ability to investigate microenvironments on spatiotemporal scales that are relevant to microorganisms themselves (Aleklett et al., 2018).

### Microarrays

**FIGURE 2:**
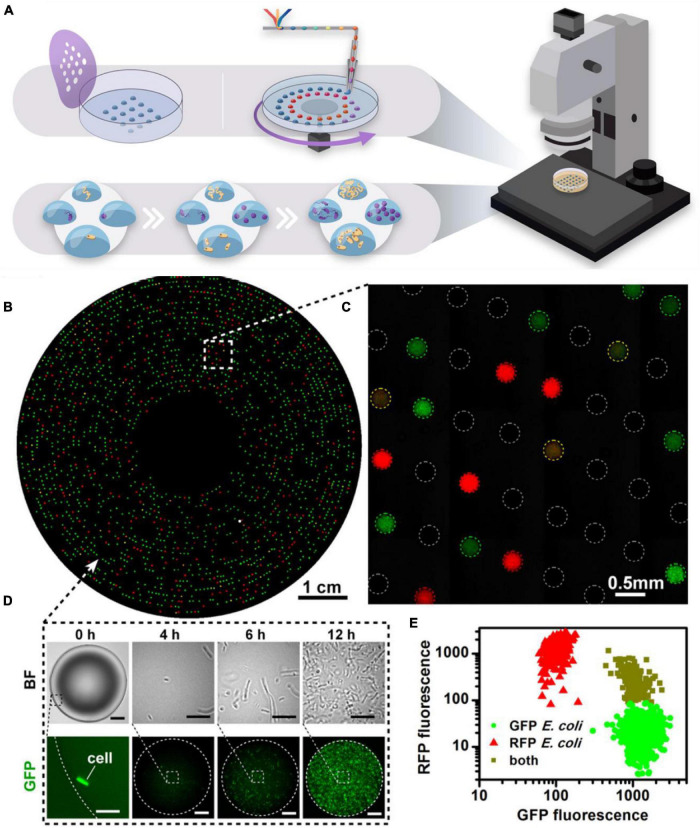
Microarray technology. **(A)** Microarrays may be stamped or written onto Petri dishes with a microfluidic pen. The hydrophilic droplets containing the cell inoculum are immersed under mineral oil to prevent evaporation and droplet coalescence. Dilution-to-extinction results in compartmentalization of the cells, such that there is one cell per droplet. Competition of resources is negated, allowing slow growers to not be outcompeted. The array is highly amenable to automated screening under a microscope. **(B–E)** Microfluidic Streak Plate demonstrating isolation and cultivation of bacterial cells from a mixed consortium of RFP- and GFP-tagged *E. coli.* Cells can be imaged in real-time **(D)** to show growth dynamics within a single droplet and relative growth **(E)** of each species can be observed by quantifying fluorescence intensity of each species. Reproduced with permission (Jiang et al., 2016). Copyright 2016, American Society for Microbiology.

Microarray technology can be seen as the first imperative step in moving from macroscale cell culturing (mL) to microscale cell culturing (μL). The key advancement involves a robotic or manual arrayer that prints cells in either liquid or alginate gel droplets onto a microscope slide which is visualized under a microscope (Figure 2). The microarray results in parallel microcultures that can be screened in a highly automated and efficient manner for applications including effects of cell-culture miniaturization on phenotype, cell morphology, and growth and lag times; drug susceptibility; and cell-cell communication, making the microarray a diversely applicable tool (Ge et al., 2016). In one instance, Srinivasan et al. (2013) used microarray printing methods to print 1,200 individual cultures of *Candida albicans* “nano-biofilms” in 30 nL droplets. The “nano-biofilms” demonstrated their similarity to conventionally grown *C. albicans* biofilms in terms of morphology, architectural growth and phenotypic characteristics. The microdroplets were further subjected to antibiotic screening for drug susceptibility, showing 28 combinatorial synergistic antifungals, as well as susceptibility to three novel antifungals.

Gao et al. (2013) created a Microbe Observation and Cultivation Array (MOCA) (Figure 1), consisting of a 4 × 6 array of 1 μL culture droplets in a Petri dish. The Petri dish is plasma treated with a mask to exhibit hydrophilic spots in an equal array. When the cell culture is micropipetted onto the hydrophilic spots in the Petri dish, dominant surface tension forces govern the spontaneous entrapment of cells. The array of droplets is subsequently covered in mineral oil to prevent evaporation during the incubation period, which arguably, might introduce bias in the recovery of majority microaerophilic or anaerobic organisms, as opposed to strictly aerobic organisms (Tang, 2018). However, the parallel cultivation sampling in aqueous droplets is highly amenable to downstream (meta)-omic analyses and attempts to bridge the gap between large volumes of conventional culturing methods and the rapid miniaturization of -omic technologies (Klein and Macosko, 2017). Using this method, Gao et al. (2013) were able to culture eight distinct species from a unique marine location off the Oregon Coast; six belonging to *Pseudoalteromonas* spp., and the remaining two identified as *Shewanella* sp., and *Colweillia piezophila.*

Merging high-throughput efficiency of microfluidics with automation of microarrays, Jiang et al. (2016) created the Microfluidic Streak Plate (MSP) (Figures 1, 2B–E). In this technique, nanoliter, sessile droplets are “written” onto a Petri dish using a disc drive and microfluidic pen, creating a large array of droplets that are easily monitored optically. Much like the MOCA array, the droplets contain the cell inoculum which are immersed in mineral oil to prevent evaporation and coalescence of the droplets. In both cases, the nano- and microliter droplets present as discrete, “mini” agar plates, eliminating growth rate bias and interspecies competition through isolation and compartmentalization. Here, the MSP method was used for high-throughput microbial cell separation and cultivation of a soil community, targeting species that degrade polycyclic aromatic hydrocarbons (PAHs) in enzyme-based fluorescence assays. This method was particularly successful in the discovery and cultivation of several species with PAH-degrading capabilities and was also successful in isolating a previously unknown *Blastococcus* species. In another application, Zhou N. et al. (2019) modified the MSP method to cultivate the gut microbiome of *Reticulitermes chinensis* termites, leading to the successful cultivation of microbes that can metabolize lignocellulose, turning wood into biofuels. In addition, 18 novel operational taxonomic units (OTUs) were documented which were phylogenetically related to *Bukholderia, Micrococcus*, and *Dysgonomas.* The authors note the utility of this method in cultivating previously unknown microbiota, which ultimately lead to the discovery of commercially important enzymes (Zhou N. et al., 2019).

Fabrication of hydrophilic array Petri dishes negates the use of complex and expensive microfabrication processes, making the Petri dish array one of the simpler techniques for the general research community and increasing usability. Furthermore, microarrays are an efficient tool for accelerating discovery of novel metabolic processes which lead to novel drug discoveries against known ADROs. However, the array of droplets on a Petri dish presents its own disadvantages: liquid droplets are susceptible to disruption during experiments and furthermore, are limited to static interrogation (Coffey and Anderson, 2014). Although cells encapsulated in gel droplets provide a more robust environment during experiments and limit disruption, conditions for studying flow are still unmet and provide a challenge that is yet to be addressed in microarray technology (Srinivasan et al., 2013).

### Micromachined Devices

We have characterized micromachining methods as a separate category to bring light to the microfabrication methods available in novel micro-culturing designs. Characteristic features of this subgroup of miniaturized culturing systems include amenability to automation and the ability to culture devices in native environments. Microfabrication offers accuracy and precision, producing grid formats that are highly predictable and that can be automated through available software for growth scoring (Ingham et al., 2007). Furthermore, several materials have been explored for the manufacture of these devices, allowing researchers to control material bulk and surface properties relating to mechanical robustness, permeability, biocompatibility, and surface charge, all of which show strong variability at the microscopic level (Wondraczek et al., 2019). An impertinent benefit of such microdevices is their portability, making natural environments outside of the lab accessible with *in situ* incubation. Thus, we can now rely on the natural microcosm to support the growth of novel species that were previously uncultivable, without having to design and manipulate a lab-enriched growth media (Stewart, 2012).

In the early 2000s, Kaeberlein et al. (2002) brought about the idea that theoretically, one could isolate and culture microorganisms simply by using their natural milieu without knowing the specific components required to support the growth of a particular ecosystem. Hence, came the development of their diffusion chambers: agar matrix inoculated with an environmental sample, sandwiched between two membranes having 0.03 μm pores (Bollmann et al., 2007). After inoculating the chamber, it is re-installed back into the same environmental sample for the incubation period. The diffusion chambers allow natural external growth factors from the surrounding environment to diffuse through the porous membranes which would otherwise be missing in standardized laboratory media, whilst restricting movement of captured microorganisms. The idea proved promising, exhibiting a ∼300-fold increase in isolate recovery compared to the Petri dish (Kaeberlein et al., 2002). Utilizing the idea of the diffusion chamber, a trapping device was similarly developed, whereby the bottom membrane was increased to a pore size of 0.2–0.6 μm. In contrast to the diffusion chamber, the trapping device was filled with sterile agar before being placed into a soil environment. Mobile species then colonize the agar, allowing for the cultivation of a number of rare species, including filamentous actinobacteria, well known as an important source of antibiotics (Gavrish et al., 2008).

Success of the diffusion chamber led to the development of the isolation chip, or “ichip” (Figure 1); a miniaturized array format of many diffusion chambers that would increase throughput and streamline the isolation and passaging process (Nichols et al., 2010). Dilution-to-extinction of the environmental sample is used to inoculate the ichip with an average of one cell per chamber, allowing for the growth of, preferably, monocultures such that one does not need to pick and isolate cultures by hand (Berdy et al., 2017). The ichip has been used extensively in a variety of environmental samples (seawater and soil) (Nichols et al., 2010), as well as in humans (mouth) (Sizova et al., 2012), allowing for parallel cultivation and isolation of a diverse assortment of phylogenetically novel species that have previously never been cultured with conventional methods.

**FIGURE 3:**
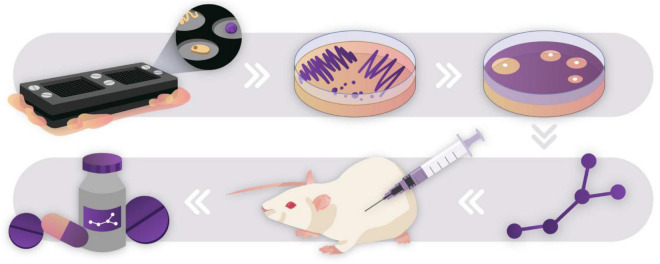
Drug discovery pipeline through culturing the “unculturable.” The ichip was used to culture novel species in an environmental soil sample (Ling et al., 2015). Species that successfully passaged onto agar plates were used to screen for antibiotic activity against *S. aureus.* Bioactive compounds were extracted, purified, characterized, and tested *in vitro* against a multitude of pathogenic organisms. This pipeline led to the discovery of teixobactin, a novel antibiotic compound that showed no mutants acquiring resistance against it in preliminary testing. After discovery, novel drugs go through rigorous safety and efficacy testing before they may be developed into safe consumer products. Novel discoveries such as this show the untapped potential of “Microbial dark matter”.

The diffusion chips and ichips described have been used on several occasions within the last few years to successfully cultivate rare environmental species, leading to the discovery of novel antibiotics including Novo10, and Neocitreamicin I and II (Sherpa et al., 2015; Lodhi et al., 2018). The isolation and passaging of a novel β-proteobacteria, *Eleftheria terrae* (Ling et al., 2015) led to the discovery of antibiotic compound teixobactin (Figure 3), which proved to have excellent antimicrobial activity against Gram-positive organisms, including drug-resistant strains. Remarkably, mutants of *Staphylococcus aureus* and *Mycobacterium tuberculosis* showed no acquired resistance to teixobactin. This discovery paints the picture that the fight against ADROs is far from over, and the ichip is a fine example of how we can harness the power of microorganisms for advances in medicine and biotechnology.

During the mid-2000s, Ingham et al. (2007) designed the micro-Petri dish: a disposable culture chip microengineered out of porous aluminum oxide (PAO) (Figure 1). The innovation behind these chips holds the same sentiment as for the ichips; however, with more focus placed on the ability to manipulate the cultivated microbes for downstream screening purposes *in situ*, including automation for ultra-high throughput efficiency. PAO is reported to be stable in a wide range of temperatures, biocompatible and inert to many solvents, allowing for the growth of a vast number of species (La Flamme et al., 2007). Furthermore, the PAO substrate contains natural pores approximately 200 nm in diameter; hence, diffusion of external factors is not limited, whilst the microbes are contained within rigid wells of the chip, with each well measuring 7 × 7 μm. The micro-Petri dish was used to show high throughput screening for (1) phenotypic variances; and (2) enzyme-based metabolic phenotypes using fluorescent assays for the targeted recovery of selected types. Isolation and recovery of species from environmental samples (Rhine river water in the Netherlands) resulted in screening more than 200,000 isolates for the targeted selection of species that perform organic phosphate degradation (Ingham et al., 2007). In comparison to standard plate culturing, the micro-Petri dish exhibited > 10-fold increase in culturability. An observable challenge with the micro-Petri dish was the contamination of monocultures via motile species or overgrowth of species; simply remedied by increasing the well size to 20 × 20 μm, thereby increasing the chamber volume. The micro-Petri dish chips result in a fast and ultra-high throughput screening system that exhibits a low degree of bias with high culturing density.

Toward the end of the decade, Jason B. Shear’s group developed a novel miniaturized device for bacterial culturing, termed “lobster traps” (Connell et al., 2010). The microbial traps are 3D-printed microcontainers, having picolitre-sized cavities (Figure 1). The walls of the lobster traps are made of photo-crosslinked Bovine Serum Albumin (BSA), which exhibits high permeability to external nutrients, metabolic waste, and other biologically relevant molecules. Although the lobster traps have not been tested for the cultivation of “unculturable” species particularly, their use has been demonstrated by growing low count/high density cell aggregates for quorum sensing studies (Connell et al., 2014). The high permeability to small molecules makes the lobster trap an enticing idea for use in the natural environment as a novel method for *in situ* incubation. The first design of the lobster traps had limited versatility; growth of a monoculture was reliant on a single motile bacteria swimming into the trap before the trap entrance was pinched off by increasing the temperature of the surrounding medium, causing the BSA walls to swell and irreversibly crosslink- a nifty feature in capturing bacteria. Subsequent designs led to printing the microchamber *in situ*, surrounding anchored, thermally set gelatin encapsulating a monoclonal culture. This results in a nested lobster trap (Connell et al., 2013), whereby one could study the intimate interactions between multiple species of cells. With this new method of printing, the group was able to show that a small aggregate of *S. aureus* exhibits heightened antibiotic resistance to β-lactams when surrounded by a culture of *Pseudomonas aeruginosa* encased within the lobster trap. Still, the group notes of potential shortcomings of the printing method, needing expensive, specialized equipment for the laser-printing, and which results in an opaque casing, making optical observations of the growing cells difficult. Furthermore, the direct writing method is not high throughput, hindering the ability to screen, isolate and culture “unculturable” environmental species (Connell et al., 2016).

For applications more specific to the human microbiome, Ismagilov’s group designed the SlipChip (Figure 1); a microfabricated device using standard photolithography and wet chemical etching techniques on soda-lime glass plates (Du et al., 2009). Two microfabricated glass plates sit atop each other, containing channels and wells in the nanoliter range that overlap when the top plate is slipped into the correct configuration. Although the design of the SlipChip lends itself well to multiplexed arrays for applications in diagnostics, parallel analytics and evaluation of contamination in various samples, a more interesting application of the SlipChip was its use in the gene-targeted isolation of a microorganism from the Human Microbiome Project’s “Most Wanted” taxa (Ma et al., 2014). A clinical sample from a human cecum was used as the inoculum, whereby stochastic confinement results in isolation of microorganisms and subsequently, pure colonies. Separating the SlipChip after cultivation splits the target colony in two; one half is used for scalable culturing whilst the other is used for quantitative PCR (qPCR) validation. This approach places emphasis on genetically targeted isolation efforts, such that minimal effort and resources are wasted isolating and cultivating off-target species. Moreover, we reinforce the notion that microdevices used for cell culturing can and should be used for complementary metagenomic studies (Rettedal et al., 2014; Versluis et al., 2019), capitalizing on the advantages of both microbiology and engineering fields to advance the discovery of novel microbial species (Emerson et al., 2017).

These novel tools for isolating and culturing microorganisms allow for exploring high-grade questions; however, they are not without their challenges. Compartmentalization of isolates is likely to introduce bias when microorganisms rely on synergistic proximity to their neighbors. For example, culturing hydrogen-producing bacteria and methanogenic archaea axenically might prove challenging for these same reasons (Berdy et al., 2017). Moreover, micro-technologies have yet to evolve to function optimally in extreme conditions; arid conditions dry out gelling agents, whereas use in sedimental aquatic environments results in anoxic conditions, limiting the number of cultivable cells. Other challenges lie in the manufacture of microfabricated devices: microfabricated chips require expensive machinery and starting materials, the expertise in know-how, as well as the diligence in precise, careful, and clean manufacturing (Whitesides, 2006). Further, many of the designed microfabricated chips are non-reusable; hence, resources may be saved in miniaturization, but manufacturing is a labor- and time-intensive process. These challenges all pose a barrier to scale-up production but help to identify where more work is needed in the field.

### Single Emulsion Droplet Microfluidics

Although there is a plethora of applications for which microfluidic chips are used, including detection and identification of microorganisms, antimicrobial susceptibility testing, microbial physiology and cell-cell dynamics, as well as applications within bacterial sensing and synthetic engineering, we will emphasize the use of droplet microfluidics for single-cell encapsulation, as we perceive this technology to be the most amenable to culturing “unculturable” and rare species, and is readily available to be integrated beyond the academic realm.

Due to the characteristic feature of laminar flow within microfluidic channels, multiphase flow is achieved that enables the generation of monodisperse droplets, commonly referred to as droplet microfluidics. Droplet microfluidics has become of particular interest for cell studies because each droplet behaves as an isolated bioreactor. With droplets ranging from pico-to-microliters, the characteristic length scales of the culturing environment is comparable to that of prokaryotic and eukaryotic cells, therefore achieving quick diffusion of gases, nutrients, metabolic waste, and the like (Aleklett et al., 2018). Droplet microfluidics are further defined by single or double emulsion droplets: single emulsion droplets refer to cells being encapsulated in an aqueous phase surrounded by an oil phase (w/o) (Figure 1); in contrast, double emulsion droplets are termed water-in-oil-in-water (w/o/w) systems, whereby cells are encapsulated in a “core” aqueous phase, surrounded by a lipophilic membrane which are dispersed in aqueous solution. Addition of a surfactant to the continuous external aqueous phase maintains stability of the droplets and inhibits coalescence (Figure 1).

Several research groups have taken advantage of microfluidics to develop novel ways in which to study microbial phenomena (Mahler et al., 2021); high-throughput procedures allow for short experimental times and significantly lowered costs. For example, Beneyton et al. (2016) used a microfluidic platform to produce nanoliter-range droplets (∼10–18 nL) at a rate of 80–90 droplets per second to screen for the enzymatic production of α-amylase by filamentous fungi, *Aspergillus niger.* In using a microfluidic platform instead of the conventional robotic microtiter plate-based platform with liquid handling systems, the group observed that screening 10^4^ variants of *A. niger* took less than 24 h and cost only $14. In comparison, they estimated that the same assay using a microtiter-plate would have taken more than 16 days and cost upwards of $8,000, based only on consumables (Beneyton et al., 2016).

In the case of single emulsions, droplets are not limited to a liquid, aqueous phase; agar droplets have also been used extensively to constrain cells. Harnessing a temperature-controlled water bath with which to bathe the syringe containing the agar ensures that the agar remains melted during the encapsulation process (Lin et al., 2011). In one application for cultivation from a marine environment, Alkayyali et al. (2021), designed the Microbe Domestication Pod (MD Pod), used to hold single-emulsion agarose beads which encapsulated marine samples for easy isolation. Once encapsulated, the gel beads are injected into the Pod, then deployed back into the environment for *in situ* cultivation. Using representative bacteria isolated from temperate marine sediment samples, the team found that the encapsulation and *in situ* cultivation led to higher metabolic activity of *Psychrobacter aquimaris* and *Bacillus licheniformis* but resulted in loss of viability of *Marinomonas polaris*. Loss of viability is attributed to the higher melting temperatures (∼45°C) required for agarose during the encapsulation process to prevent gelling (Alkayyali et al., 2021). This increased temperature is tolerable to mesophilic species, but proved detrimental to psychrotolerant species, which limits the usefulness of agarose in cultivating temperature-sensitive microorganisms (Russell, 2003). Furthermore, due to the hydrophilic nature of agarose, it has been reported that agarose microbeads are susceptible to significant swelling (Lin et al., 2011), which further alters the diffusion properties of metabolites and waste, inevitably affecting the growing cells (Chen et al., 2015).

As previously noted, culturing of many microbial species is impeded by the inability to mimic their natural environment—this may be especially true for obligate anaerobes, requiring anaerobic chambers and special treatment by pre-reducing all liquid media that is to be used in contact with anaerobes. These cumbersome efforts may be wasted during inefficient transport to and processing of samples within the anaerobic chamber, resulting in a loss of low-abundant species. Microfluidics may be used to address some of these issues, as demonstrated by Villa et al. (2019). The group developed the MicDrop, a droplet microfluidic platform that was used to culture human gut microbiota successfully in an anaerobic chamber. The water-in-oil droplets resulted in 2.6 times higher diversity than when samples were grown in mixed conditions and were simultaneously combined with molecular techniques to validate growth of isolates in the microfluidic droplets. This platform demonstrates the utility of droplet microfluidics in traditionally difficult culturing settings (Villa et al., 2019).

Much like micromachined devices, a major challenge in using microfluidics is accessibility to expensive fabrication equipment. Most microfluidic devices are produced by casting a mold of poly(dimethylsiloxane) (PDMS); however, the mold must first be designed and manufactured with expensive and brittle silicon wafers (Weibel et al., 2007). Microelectromechanical systems (MEMS) microfabrication techniques offer several ways to etch, emboss or lithograph silicon molds. One of the more frequently used fabrication methods now is soft photolithography; a technique developed by Xia and Whitesides (1998). Soft lithography, although still making use of silicon wafers for initial fabrication, uses photo-crosslinkable polymers to create a master mold of the microfluidic device required. The designed master can subsequently be used repeatedly to cast microfluidic devices in PDMS, a quick and straightforward process to do in any lab. PDMS, in and of itself, has attractive properties for use in microfluidics due to its optical transparency, biocompatibility, permeability to gases, and low cost. However, fabrication of the device mold still requires use of a clean room and the extensive “know-how,” an obstacle for many researchers. For those who do not have access to such resources, one can buy commercialized microfluidic chips, significantly reducing the amount of time designing and fabricating one’s own chips. However, commercialized chips may have limited applicability for explorative studies, particularly for environmental samples. To increase accessibility to and personalization of the fabrication process, several public foundries have been developed at universities that allow users to send in their own designs for microfabricated chips, negating the investment of time and capital for users who do not want to be specifically trained in the process (Weibel et al., 2007).

### Double Emulsion Droplet Microfluidics and Polymer-Based Nanocultures

In a similar fashion to single emulsion droplets, double emulsion droplets are generated with hydrodynamic pressure flow and co-flowing geometry within microfluidic channels. In contrast is the interphase at the channel junction which consists of three phases. The innermost phase, or core, is an aqueous phase containing the cell inoculum. The middle phase, hydrophobic in nature, may be polymeric or oleophilic, forming double emulsion droplets which are suspended in a continuous aqueous (hydrophilic) phase, including a surfactant to stabilize the droplets in solution. The addition of a membrane to house the encapsulated cells imparts unique functionality to the droplets, such as mechanical robustness for long-term studies, optical transparency for microscopy, and semi-permeability such that diffusion of chemical species can be selectively controlled (Raj and Chakraborty, 2020).

High-throughput assays may be achieved with droplet microfluidics; whereby double emulsion droplets compartmentalize chemical reactions into nanoliter-scale bioreactors. The chemical assay may then be complemented with well-established sorting methods, such as fluorescence-activated cell sorting (FACS), for the discrimination of successful assay products. Zinchenko et al. (2014) has demonstrated the powerful utility of this platform, screening for enriched cellular clones that produce catalytically active enzymes from as many as 10^6^ low-active variants, all encapsulated in 10 μm droplets. Moreover, the group was able to show that the assay can be heat inactivated to stop catalytic function, and furthermore, the droplets can be successfully frozen (−80°C) and subsequently thawed for discontinuous workflows. Discontinuous workflows could prove hugely advantageous for breaking up long workflows into shorter time-frames, allowing for more flexibility for researchers.

In applications requiring more robust microcapsules for use in diverse environments, a polymeric membrane provides the means to constrain the cells in a tough shell, without impeding permeability of small chemical species. For example, Barlow et al. (2017) used diblock co-polymer poly(ethylene glycol)-*b*- poly(D,L-lactic acid) (mPEG-PGDLLA) to generate double emulsion polymersomes with encapsulated *Bacillus subtilis* for the remediation of elemental selenium from wastewater. The polymersomes demonstrated that encapsulated *B. subtilis* remained viable and produced robust biofilm within the microcapsules, reducing soluble selenite (Na_2_SeO_3_) into less toxic elemental selenium (Se) (Barlow et al., 2017). In this case, the polymeric membrane is biodegradable, therefore, harvesting the microcapsule contents becomes simple. The polymersomes are versatile and allow for remediation of polluted areas that are not amenable to traditional wastewater treatment.

**FIGURE 4:**
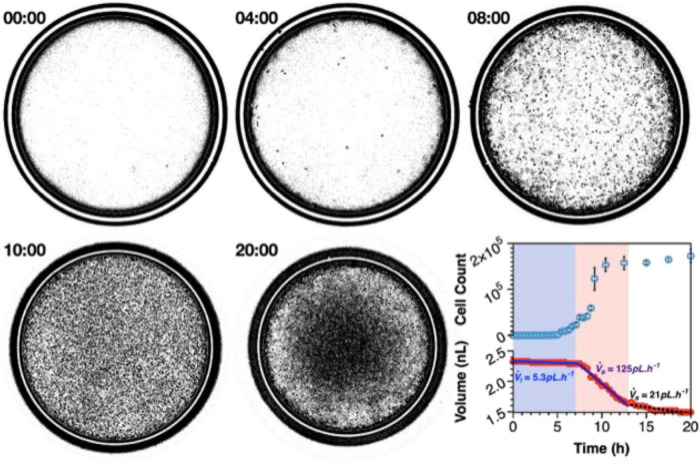
Nanocultures provide an ideal environment to study microbial growth dynamics over spatial-temporal scales that recapitulate that of macroscale flask cultures. Growth of *P. aeruginosa* was observed over the course of 20 h, whereby exponential growth is achieved between 7 and 13 h and stationary phase is attained after 13 h of incubation. The nanocultures shrink in size due to consumption of resources, and the volumetric flowrate of water leaving the capsules may be calculated at each stage by observing the decrease in capsule diameter in real-time (Usman et al., 2021).

In our own novel application for double emulsion microdroplets, the Niepa group has designed nanocultures: nanoliter-sized capsules that contain nutrient broth and grow microorganisms inside planktonically, with each capsule serving as its own, miniaturized flask culture (Niepa and Davidson, 2020; Usman et al., 2021). An example of this is shown in Figure 4, whereby nanocultures were generated with *P. aeruginosa* (PAO1) and growth dynamics of the monoculture were observed in real-time over a course of 20 h (Usman et al., 2021). Planktonic growth of the PAO1 cells simulated accurately the same planktonic growth that is commonly seen in macroscale flask cultures. Furthermore, the nanocultures shrank significantly over the course of exponential growth, which can be attributed to resource consumption and subsequent osmosis to maintain osmotic equilibrium over the capsule membrane (Chang et al., 2015). It is possible then, to realize that nanocultures present an accessible tool for long-term studies, whereby cells may change phylogenetically due to various stressors including loss of space and competition for resources. Population heterogeneity may be introduced in this way, such as with persister cells, and their microbial dynamics assessed under a microscope. The nanocultures demonstrate their use in applications for observing growth of microbial consortia in a high throughput manner for spatial and temporal dynamics.

Microcapsules provide an ideal environment for the study of microbial communities that can be finely tuned and controlled to study the effects of independent stimuli, making it easy to decouple between physico-chemical dynamics. Furthermore, the polymeric membrane can be manipulated to satisfy the specific design requirements for varying applications. For example, the size of the nanocultures is controlled by both physical and chemical means: changing flowrates of the liquid phases present physical means to change capsule size during the encapsulation process, or a difference in osmotic pressures may be used to either draw water in or out of the capsules after their collection (Usman et al., 2021). This changes the concentration of chemical species within the capsule, and further dictates the success at which inter-cellular communication occurs. Although seemingly trivial, many cell functions are governed by community signaling and synergistic growth of recalcitrant species may heavily rely on such signaling (Park et al., 2011; Cai et al., 2019). Moreover, materials such as PDMS allow for chemical functionalization to control diffusive and mechanical properties of the nanocultures. In an explorative study, Manimaran et al. (2020) designed a new polymeric biomaterial for the use of cell encapsulation. Like commercial PDMS, the novel polymer is a mix of vinyl and hydromethylsiloxane polymers with the addition of N, N-dimethylallylamine (DMAA). The addition of DMAA into the polymer prevents tight crosslinking, subsequently resulting in a polymer network with a larger free volume. This should, in turn, increase the permeability of the membrane—these studies are ongoing.

One of the characteristics of PDMS is its mechanical robustness and elasticity, exhibiting a Young’s modulus of ∼0.5–3 MPa (Wang et al., 2014). Although beneficial for creating robust capsules for cell encapsulation, it becomes a challenge for downstream processing, which includes breaking the capsules open to retrieve the contents for further study. Therefore, one of the key benefits of functionalizing the polymeric membrane is the ability to reduce the Young’s modulus, therefore, creating microcapsules that are more brittle and that require less shear force to lyse the capsules, done simply with sonication or mechanical bead beating.

As discussed previously, a major improvement in the miniaturization of culturing is the ability to incubate samples *in situ*, as with the ichip. Hence, it is important that the nanocultures described here comply with this design requirement too, to study the effect of unknown metabolites on environmental microcosms (Barkal et al., 2016). A challenge presents itself here in the retrieval of nanocultures after their dissemination into the environment, due to the free-floating nature of each nanoculture. To this end, the PDMS membrane may be functionalized with ferrimagnetic iron oxide (Fe_3_0_4_), thereby allowing the nanocultures to be collected by simply moving a magnet nearby the sample. Therefore, the nanocultures may be suspended freely without confining them to a substrate, which might result in less effective diffusion of metabolites across the membrane.

## Applications of Microbial-Based Microsystems and Perceived Challenges

**FIGURE 5:**
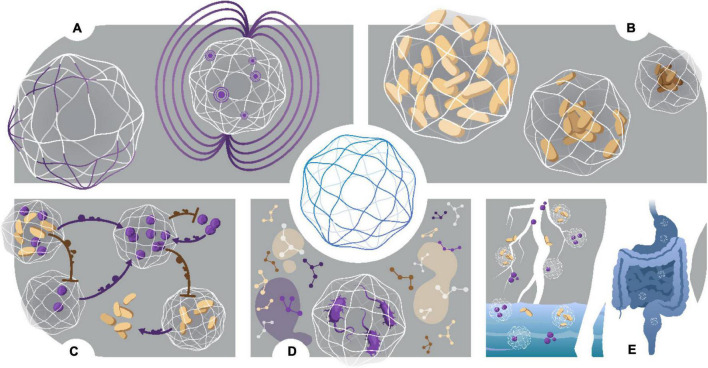
Characterization and applications of nanocultures. **(A)** Chemical and Magnetic functionalization. Nanocultures are custom designed to fit specific applications. Addition of functional group DMAA into the polymer membrane increases free volume, changing the selective permeability properties of the membrane. Similarly, addition of magnetic oxide allows for easy retrieval after nanocultures are freely suspended in an environmental sample. To collect nanocultures, a magnet is simply moved over the sample. **(B)** Investigation of osmotic stress. Nanocultures may be used to study single cell and/or community response to physical insults, such as osmotic stress. **(C)** Biochemical Interactions. Intra- and Inter-species dynamics may be studied in real time, whereby the semi-permeable membrane provides physical containment of the cells but allows for cross signaling between nanocultures in the form of small molecules. Furthermore, secreted small molecules may be studied for biological relevance, such as drug discovery or beneficial secondary metabolites for symbiotic relationships. **(D)** Growth of fastidious species. Growth of fastidious species has been demonstrated by culturing *C. difficile* under a microscope, negating the use of anaerobic jars or chambers. **(E)** Culturing the “unculturable.” Nanocultures can be used to successfully culture the “unculturable” from many environmental sources, such as soil, marine and human microbiomes.

Engineered microsystems are an ideal way to miniaturize culturing of microorganisms from a myriad of environments (Figure 5). Materials that provide a selectively permeable, but protective environment for isolated, or co-cultured species to grow whilst removing competition for resources. This is especially beneficial for slow growing microorganisms that are normally outcompeted by fast-growing species. Dilution-to-extinction provides a controlled way to serially dilute samples to a point of one cell per compartment average, the importance of which is to study single-cell dynamics in a confined environment. Isolated colonies can be probed with chemical stimuli, and phylogenetic responses may be observed in optically transparent systems. More complex consortia can also be observed for the purpose of studying intra-and inter-species relationships; an important aspect in defining both symbiotic and antagonistic behaviors between species, as well as pathogenic switching in the case of opportunistic pathogens. Study of complex relationships between species will help us further understand the use of secondary metabolites within an ecosystem, with the discovery of novel antimicrobials, as well as important enzymes, dependent on these inter-species dynamics. Moreover, many recalcitrant and fastidious species require secondary metabolites that are not fully known or understood (Nichols et al., 2008). With *in situ* incubation, having to understand the detailed and complex requirements for nutrients of all distinct species is negated, whilst the natural milieu provides the nutrients to stimulate cultivation of “unculturable” species. Engineered microsystems also allow for the cultivation of microorganisms in traditionally difficult conditions. For example, droplet microfluidics allow for simulating hypoxic environments without the use of anaerobic chambers or jars, significantly reducing footprint as well as increasing accessibility to and manipulation of the anaerobic environment. To demonstrate this, polymeric nanocultures were used to successfully grow *Clostridium difficile*, an obligate anaerobe, without the use of anaerobic jars or a chamber (Niepa and Davidson, 2020). *Clostridium difficile* has been noted as an “Urgent Threat” on the US CDC 2019 report on Antimicrobial Resistance Threats (CDC, 2020). Hence, nanocultures can provide a novel method for studying complex interactions of gut microbiota against *C. difficile* in a hypoxic environment, whilst remaining optically accessible under a microscope. This allows for the study of microorganisms in real time, which can further be complemented with -omics studies, providing detailed analysis of population dynamics (who is there, and in what abundance?), as well as which specific genes are upregulated or downregulated for species fitness, and further, which type of metabolites are being produced. These factors can significantly alter microcosms and ultimately lead to environmental and host colonization in deterministic patterns.

These microsystems are not only limited to the isolation and cultivation of “unculturable” species. The advent of miniaturizing culturing techniques has been an inspiration for many applications we can now achieve with the control, precision, and resolution needed to identify targets of study (Hu et al., 2021). In biotechnology industries, drug and enzymatic metabolite screening is typically associated with low-throughput, high-cost methods (Boedicker et al., 2008). Microfluidics improves on these methods by significantly scaling down and miniaturizing every step of the process. Results are achieved within a few hours as opposed to days, which ultimately drives down the cost of screening processes. Within the pharmaceutical industry, diagnostic tools, biologic assays, and analytical tools are desperately needed to drive discovery and productivity (Li et al., 2017); microfluidics offer high replicability and automation. Furthermore, lab-on-a-chip (LOC) devices offer predictive tools for how effectively a therapeutic performs on human cells, while at the same time monitors safety and toxicity (Lee et al., 2016). These devices can be further used to study prokaryotic-eukaryotic interactions (Zhou W. et al., 2019) and associated phenomena (Andersson and van den Berg, 2004), such as pathogenic switches (Son et al., 2015), biofilm formation (Song et al., 2014; Chang et al., 2015; Zhang et al., 2019; Coenye et al., 2020) and immunogenicity (Poceviciute and Ismagilov, 2019), for example. In biomedical industries, another much needed application for microfluidics is in the use of medical devices, such as POC analytic tests (Chin et al., 2011). Microfluidics may prove an effective solution, lending itself to small sample volumes, use by untrained personnel and low cost (Warren et al., 2014). Therefore, the microsystems discussed here may be used to firstly, cultivate species from a myriad of novel microbiomes, and subsequently, discover and test novel metabolites and enzymes with high-throughput efficiency, advancing precision medicine and commercial fields alike (Figure 5). As we understand more about our unique microbiomes and how they affect their host environments, it becomes imperative to understand how to modulate and treat dysbiosis within these microbiomes; a deep understanding of microbial interactions is needed to achieve this.

With all the possible applications achievable with microfluidics, it is not to say that the field is without its challenges. For now, the field at large has remained mostly proof-of-concept within the academic realm. For most of these applications to be realized, much work needs to be done in commercialization. Furthermore, microfluidics must prove successful in these varying applications before they may become practical, standardized technologies that are inexpensively available to everyone, including developing economies (Orive et al., 2004).

More specific challenges pertaining to device fabrication include materials design. For example, as discussed in Aleklett et al. (2018), use of PDMS in microfluidic devices to simulate natural environments is somewhat unnatural; as an intrinsically hydrophobic elastomer, it does not offer a heterogeneous environment that includes mechanical manipulation for cells, such that natural environments do. PDMS can be treated with oxygen plasma to induce hydrophilic surface changes, but this is a temporary solution, as movement of oligomers within the PDMS network tend to return the surface to its lesser free energy state (Lee et al., 2003). With fixtures in custom device design, some of these challenges are mitigated with the addition of polydisperse nanoparticles, or flexible pillars, simulating a more natural and complex habitat and introducing the ability to study mechano-microbiology (Dufrêne and Persat, 2020) in addition to chemotaxis.

## Conclusion and Outlook

The miniaturization of culturing methods affords exciting and novel designs to study “microbial dark matter” and their associated microbial dynamics. Microfabrication processes have resulted in intricate devices that interrogate the immediate microenvironment of cells, leaving behind conventional nutrient-rich broth in exchange for the natural milieu found all around us. Using microfabricated devices to discover novel metabolic pathways will usher in a new generation of antibiotics, POC diagnostics, high-throughput screening and many other useful inventions. However, work needs to be done to make the microfabrication processes more accessible to researchers who are not well-versed in clean-room fabrication methods (Morgan et al., 2016). Microfabrication resources and equipment present a large barrier in scalability, as resources are expensive and not easy to use, hindering much needed development in the field (Zinchenko et al., 2014).

Questions regarding microorganisms and their interactions are constantly evolving to reflect changes in what we know and similarly, methods in how we study these unique microsystems require change too. Hence, the ability to control tunable properties surrounding the study of microbial spaces and at the appropriate scale is exactly what micro-technologies can offer us, with precision and reproducibility. These are generally low-cost technologies that bridge the gap between microbiology and engineering, and now is a perfect time to embrace the abilities of the differing fields to explore and discover, just what lies beneath in the expansive microverse of “Microbial Dark Matter.”

## Author Contributions

S-LD and THRN conceived, wrote the review, and designed the figures. Both authors have critically reviewed and given approval to the final version of the manuscript.

## Conflict of Interest

The authors declare that the research was conducted in the absence of any commercial or financial relationships that could be construed as a potential conflict of interest.

## Publisher’s Note

All claims expressed in this article are solely those of the authors and do not necessarily represent those of their affiliated organizations, or those of the publisher, the editors and the reviewers. Any product that may be evaluated in this article, or claim that may be made by its manufacturer, is not guaranteed or endorsed by the publisher.

## References

[B1] Aleklett K. Kiers E. T. Ohlsson P. Shimizu T. S. Caldas V. E. A. Hammer E. C. (2018). Build your own soil: exploring microfluidics to create microbial habitat structures. *ISME J.* 12 312–319. 10.1038/ismej.2017.184 29135971 PMC5776464

[B2] Alkayyali T. Pope E. Wheatley S. K. Cartmell C. Haltli B. Kerr R. G. (2021). Development of a microbe domestication pod (MD Pod) for in situ cultivation of micro-encapsulated marine bacteria. *Biotechnol. Bioeng.* 118 1166–1176. 10.1002/bit.27633 33241862

[B3] Andersson H. van den Berg A. (2004). Microtechnologies and nanotechnologies for single-cell analysis. *Curr. Opin. Biotechnol.* 15 44–49. 10.1016/j.copbio.2004.01.004 15102465

[B4] Banks P. (2009). *The Microplate Market Past, Present and Future [Online]. Drug Discovery World.* Available online at: https://www.ddw-online.com/the-microplate-market-past-present-and-future-1127-200904/ (accessed 01 04, 2021)

[B5] Barkal L. J. Theberge A. B. Guo C.-J. Spraker J. Rappert L. Berthier J. (2016). Microbial metabolomics in open microscale platforms. *Nat. Commun.* 7:10610. 10.1038/ncomms10610 26842393 PMC4742997

[B6] Barlow J. Gozzi K. Kelley C. P. Geilich B. M. Webster T. J. Chai Y. (2017). High throughput microencapsulation of Bacillus subtilis in semi-permeable biodegradable polymersomes for selenium remediation. *Appl. Microbiol. Biotechnol.* 101 455–464. 10.1007/s00253-016-7896-7 27744558 PMC5203941

[B7] Beneyton T. Wijaya I. P. M. Postros P. Najah M. Leblond P. Couvent A. (2016). High-throughput screening of filamentous fungi using nanoliter-range droplet-based microfluidics. *Sci. Rep.* 6:27223. 10.1038/srep27223 27270141 PMC4895158

[B8] Berdy B. Spoering A. L. Ling L. L. Epstein S. S. (2017). In situ cultivation of previously uncultivable microorganisms using the ichip. *Nat. Protoc.* 12 2232–2242. 10.1038/nprot.2017.074 29532802

[B9] Bodor A. Bounedjoum N. Vincze G. E. Erdeiné Kis Á Laczi K. Bende G. (2020). Challenges of unculturable bacteria: environmental perspectives. *Rev. Environ. Sci. Biotechnol.* 19 1–22. 10.1007/s11157-020-09522-4

[B10] Boedicker J. Q. Li L. Kline T. R. Ismagilov R. F. (2008). Detecting bacteria and determining their susceptibility to antibiotics by stochastic confinement in nanoliter droplets using plug-based microfluidics. *Lab Chip* 8 1265–1272. 10.1039/b804911d 18651067 PMC2612531

[B11] Bollmann A. Lewis K. Epstein S. S. (2007). Incubation of environmental samples in a diffusion chamber increases the diversity of recovered isolates. *Appl. Environ. Microbiol.* 73 6386–6390. 10.1128/AEM.01309-07 17720826 PMC2075052

[B12] Cai P. Sun X. Wu Y. Gao C. Mortimer M. Holden P. A. (2019). Soil biofilms: microbial interactions, challenges, and advanced techniques for ex-situ characterization. *Soil Ecol. Lett.* 1 85–93.

[B13] CDC (2020). *Antibiotic Resistance Threats in the United States 2019.* Atlanta, GA: CDC.

[B14] Chang C. B. Wilking J. N. Kim S.-H. Shum H. C. Weitz D. A. (2015). Monodisperse emulsion drop microenvironments for bacterial biofilm growth. *Small* 11 3954–3961. 10.1002/smll.201403125 25959709

[B15] Chaudhary D. K. Khulan A. Kim J. (2019). Development of a novel cultivation technique for uncultured soil bacteria. *Sci. Rep.* 9:6666. 10.1038/s41598-019-43182-x 31040339 PMC6491550

[B16] Chen Y. Liu G.-T. Xu J.-H. Luo G.-S. (2015). The dynamic mass transfer of surfactants upon droplet formation in coaxial microfluidic devices. *Chem. Eng. Sci.* 132 1–8. 10.1016/j.ces.2015.04.006

[B17] Chin C. D. Laksanasopin T. Cheung Y. K. Steinmiller D. Linder V. Parsa H. (2011). Microfluidics-based diagnostics of infectious diseases in the developing world. *Nat. Med.* 17 1015–1019. 10.1038/nm.2408 21804541

[B18] Coenye T. Kjellerup B. Stoodley P. Bjarnsholt T. (2020). The future of biofilm research – Report on the ‘2019 Biofilm Bash’. *Biofilm* 2:100012. 10.1016/j.bioflm.2019.100012 33447799 PMC7798458

[B19] Coffey B. M. Anderson G. G. (2014). Biofilm formation in the 96-well microtiter plate. *Methods Mol. Biol.* 1149 631–641. 10.1007/978-1-4939-0473-0_48 24818938

[B20] Connell J. Ritschdorff E. Shear J. (2016). 3D printing of photoresponsive biomaterials for control of bacterial microenvironments. *Anal. Chem.* 88 12264–12271. 10.1021/acs.analchem.6b03440 27782402

[B21] Connell J. L. Kim J. Shear J. B. Bard A. J. Whiteley M. (2014). Real-time monitoring of quorum sensing in 3D-printed bacterial aggregates using scanning electrochemical microscopy. *Proc. Natl. Acad. Sci. U.S.A.* 111 18255–18260. 10.1073/pnas.1421211111 25489085 PMC4280622

[B22] Connell J. L. Ritschdorff E. T. Whiteley M. Shear J. B. (2013). 3D printing of microscopic bacterial communities.. *Proc. Natl. Acad. Sci. U.S.A.* 110 18380–18385. 10.1073/pnas.1309729110 24101503 PMC3832025

[B23] Connell J. L. Wessel A. K. Parsek M. R. Ellington A. D. Whiteley M. Shear J. B. (2010). Probing prokaryotic social behaviors with bacterial “lobster traps”. *mBio* 1:e00202–00210. 10.1128/mBio.00202-10 21060734 PMC2975351

[B24] Dance A. (2020). The search for microbial dark matter. *Nature (London)* 582 301–303. 10.1038/d41586-020-01684-z 32514023

[B25] Du W. Li L. Nichols K. P. Ismagilov R. F. (2009). SlipChip. *Lab Chip* 9 2286–2292. 10.1039/b908978k 19636458 PMC2719824

[B26] Dufrêne Y. F. Persat A. (2020). Mechanomicrobiology: how bacteria sense and respond to forces. *Nat. Rev. Microbiol.* 18 227–240. 10.1038/s41579-019-0314-2 31959911

[B27] Emerson J. B. Adams R. I. Román C. M. B. Brooks B. Coil D. A. Dahlhausen K. (2017). Schrödinger’s microbes: tools for distinguishing the living from the dead in microbial ecosystems. *Microbiome* 5:86. 10.1186/s40168-017-0285-3 28810907 PMC5558654

[B28] Franklin M. J. Chang C. Akiyama T. Bothner B. (2015). New technologies for studying biofilms. *Microbiol. Spectr.* 3:10. 10.1128/microbiolspec.MB-0016-2014 26350329 PMC4821632

[B29] Gao W. Navarroli D. Naimark J. Zhang W. Chao S.-H. Meldrum D. R. (2013). Microbe observation and cultivation array (MOCA) for cultivating and analyzing environmental microbiota. *Microbiome* 1:4. 10.1186/2049-2618-1-4 24468000 PMC3869193

[B30] Gavrish E. Bollmann A. Epstein S. Lewis K. (2008). A trap for in situ cultivation of filamentous actinobacteria. *J. Microbiol. Methods* 72 257–262. 10.1016/j.mimet.2007.12.009 18255181 PMC2293972

[B31] Ge Z. Girguis P. R. Buie C. R. (2016). Nanoporous microscale microbial incubators. *Lab Chip* 16 480–488. 10.1039/c5lc00978b 26584739

[B32] Göing S. Jung K. (2021). Viable but nonculturable gastrointestinal bacteria and their resuscitation. *Arch. Gastroenterol. Res.* 2:7.

[B33] Hansen R. H. Timm A. C. Timm C. M. Bible A. N. Morrell-Falvey J. L. Pelletier D. A. (2016). Stochastic assembly of bacteria in microwell arrays reveals the importance of confinement in community development. *PLoS One* 11:e0155080. 10.1371/journal.pone.0155080 27152511 PMC4859483

[B34] Hu B. Xu P. Ma L. Chen D. Wang J. Dai X. (2021). One cell at a time: droplet-based microbial cultivation, screening and sequencing. *Mar. Life Sci. Technol.* 3 169–188. 10.1007/s42995-020-00082-8 PMC10077293 37073344

[B35] Ingham C. J. Sprenkels A. Bomer J. Molenaar D. Van Den Berg A. Van Hylckama Vlieg J. E. (2007). The micro-Petri dish, a million-well growth chip for the culture and high-throughput screening of microorganisms. *Proc. Natl. Acad. Sci. U.S.A.* 104 18217–18222. 10.1073/pnas.0701693104 17989237 PMC2084323

[B36] Jiang C.-Y. Dong L. Zhao J.-K. Hu X. Shen C. Qiao Y. (2016). High-throughput single-cell cultivation on microfluidic streak plates. *Appl. Environ. Microbiol.* 82:2210. 10.1128/AEM.03588-15 26850294 PMC4807504

[B37] Kaeberlein T. Lewis K. Epstein S. S. (2002). Isolating “uncultivable” microorganisms in pure culture in a simulated natural environment. *Science* 296 1127–1129. 10.1126/science.1070633 12004133

[B38] Klein A. M. Macosko E. (2017). InDrops and Drop-seq technologies for single-cell sequencing. *Lab Chip* 17 2540–2541. 10.1039/c7lc90070h 28721415

[B39] La Flamme K. E. Popat K. C. Leoni L. Markiewicz E. La Tempa T. J. Roman B. B. (2007). Biocompatibility of nanoporous alumina membranes for immunoisolation. *Biomaterials* 28 2638–2645. 10.1016/j.biomaterials.2007.02.010 17335895 PMC3225223

[B40] Lee J. N. Park C. Whitesides G. M. (2003). Solvent compatibility of poly(dimethylsiloxane)-based microfluidic devices. *Anal. Chem.* 75 6544–6554. 10.1021/ac0346712 14640726

[B41] Lee S. H. Ha S. K. Choi I. Choi N. Park T. H. Sung J. H. (2016). Microtechnology-based organ systems and whole-body models for drug screening. *Biotechnol. J.* 11 746–756. 10.1002/biot.201500551 27125245

[B42] Li Y. Yang X. Zhao W. (2017). Emerging microtechnologies and automated systems for rapid bacterial identification and antibiotic susceptibility testing. *Slas Technol.* 22 585–608. 10.1177/2472630317727519 28850804 PMC5835395

[B43] Lin Y. S. Yang C. H. Lu K. Huang K. S. Zheng Y. Z. (2011). Synthesis of agar microparticles using temperature-controlled microfluidic devices for *Cordyceps militaris* cultivation. *Electrophoresis* 32 3157–3163. 10.1002/elps.201100343 22012813

[B44] Ling L. L. Schneider T. Peoples A. J. Spoering A. L. Engels I. Conlon B. P. (2015). A new antibiotic kills pathogens without detectable resistance. *Nature* 517 455–459. 25561178 10.1038/nature14098 PMC7414797

[B45] Locey K. J. Lennon J. T. (2016). Scaling laws predict global microbial diversity. *Proc. Natl. Acad. Sci. U.S.A.* 113 5970–5975. 10.1073/pnas.1521291113 27140646 PMC4889364

[B46] Lodhi A. F. Zhang Y. Adil M. Deng Y. (2018). Antibiotic discovery: combining isolation chip (iChip) technology and co-culture technique. *Appl. Microbiol. Biotechnol.* 102 7333–7341. 10.1007/s00253-018-9193-0 29974183

[B47] Lok C. (2015). Mining the microbial dark matter. *Nature (London)* 522 270–273. 10.1038/522270a 26085253

[B48] Ma L. Kim J. Hatzenpichler R. Karymov M. A. Hubert N. Hanan I. M. (2014). Gene-targeted microfluidic cultivation validated by isolation of a gut bacterium listed in human microbiome project's Most Wanted taxa. *Proc. Natl. Acad. Sci. U.S.A.* 111:9768. 10.1073/pnas.1404753111 24965364 PMC4103313

[B49] Mahler L. Niehs S. P. Martin K. Weber T. Scherlach K. Hertweck C. (2021). Highly parallelized droplet cultivation and prioritization of antibiotic producers from natural microbial communities. *eLife* 10:e64774. 10.7554/eLife.64774 33764297 PMC8081529

[B50] Manimaran N. H. Usman H. Kamga K. L. Davidson S.-L. Beckman E. Niepa T. H. (2020). Developing a functional poly (dimethylsiloxane)-based microbial nanoculture system using dimethylallylamine. *ACS Appl. Mater. Interfaces* 12 50581–50591. 10.1021/acsami.0c11875 33119264

[B51] Morgan A. J. L. Hidalgo San Jose L. Jamieson W. D. Wymant J. M. Song B. Stephens P. (2016). Simple and versatile 3D printed microfluidics using fused filament fabrication. *PLoS One* 11:e0152023. 10.1371/journal.pone.0152023 27050661 PMC4822857

[B52] Nichols D. Cahoon N. Trakhtenberg E. M. Pham L. Mehta A. Belanger A. (2010). Use of Ichip for high-throughput in situ cultivation of “uncultivable” microbial species. *Appl. Environ. Microbiol.* 76 2445–2450. 10.1128/aem.01754-09 20173072 PMC2849220

[B53] Nichols D. Lewis K. Orjala J. Mo S. Ortenberg R. Connor P. (2008). Short peptide induces an “uncultivable” microorganism to grow in vitro. *Appl. Environ. Microbiol.* 74 4889. 10.1128/AEM.00393-08 18515474 PMC2519364

[B54] Niepa T. H. R. Davidson S.-L. (2020). *Microcapsules and Methods of Using the Same.* U.S. Patent Application 2020/0108021A1.

[B55] Niepa T. H. R. Hou L. Jiang H. Goulian M. Koo H. Stebe K. J. (2016). Microbial nanoculture as an artificial microniche. *Sci. Rep.* 6:30578. 27476816 10.1038/srep30578 PMC4967889

[B56] Orive G. Maria Hernández R. RodríGuez Gascón A. Calafiore R. Swi Chang T. M. Vos P. D. (2004). History, challenges and perspectives of cell microencapsulation. *Trends Biotechnol.* 22 87–92. 10.1016/j.tibtech.2003.11.004 14757043

[B57] Park J. Kerner A. Burns M. A. Lin X. N. (2011). Microdroplet-enabled highly parallel co-cultivation of microbial communities. *PLoS One* 6:e17019. 10.1371/journal.pone.0017019 21364881 PMC3045426

[B58] Pienaar J. A. Singh A. Barnard T. G. (2016). The viable but non-culturable state in pathogenic *Escherichia coli*: a general review. *Afr. J. Lab.Med.* 5 1–9. 10.4102/ajlm.v5i1.368 28879110 PMC5436400

[B59] Poceviciute R. Ismagilov R. F. (2019). Human-gut-microbiome on a chip. *Nat. Biomed. Eng.* 3 500–501. 10.1038/s41551-019-0425-0 31278388

[B60] Raj M. K. Chakraborty S. (2020). PDMS microfluidics: a mini review. *J. Appl. Polym. Sci.* 137:48958. 10.1002/app.48958

[B61] Ramamurthy T. Ghosh A. Pazhani G. P. Shinoda S. (2014). Current perspectives on viable but non-culturable (VBNC) pathogenic bacteria. *Front. Public Health* 2:103. 10.3389/fpubh.2014.00103 25133139 PMC4116801

[B62] Rettedal E. A. Gumpert H. Sommer M. O. A. (2014). Cultivation-based multiplex phenotyping of human gut microbiota allows targeted recovery of previously uncultured bacteria. *Nat. Commun.* 5:4714. 10.1038/ncomms5714 25163406

[B63] Russell A. D. (2003). Lethal effects of heat on bacterial physiology and structure. *Sci. Prog.* 86 115–137. 10.3184/003685003783238699 12838607 PMC10368340

[B64] Sherpa R. T. Reese C. J. Montazeri Aliabadi H. (2015). Application of iChip to grow “Uncultivable” microorganisms and its impact on antibiotic discovery. *J. Pharm. Pharm. Sci.* 18 303–315. 10.18433/j30894 26517134

[B65] Sizova M. V. Hohmann T. Hazen A. Paster B. J. Halem S. R. Murphy C. M. (2012). New approaches for isolation of previously uncultivated oral bacteria. *Appl. Environ. Microbiol.* 78 194–203. 10.1128/AEM.06813-11 22057871 PMC3255620

[B66] Son K. Brumley D. R. Stocker R. (2015). Live from under the lens: exploring microbial motility with dynamic imaging and microfluidics. *Nat. Rev. Microbiol.* 13 761–775. 10.1038/nrmicro3567 26568072

[B67] Song J. L. Au K. H. Huynh K. T. Packman A. I. (2014). Biofilm responses to smooth flow fields and chemical gradients in novel microfluidic flow cells. *Biotechnol. Bioeng.* 111 597–607. 10.1002/bit.25107 24038055 PMC3910156

[B68] Srinivasan A. Leung K. P. Lopez-Ribot J. L. Ramasubramanian A. K. (2013). High-throughput nano-biofilm microarray for antifungal drug discovery. *mBio* 4:e00331-00313. 10.1128/mBio.00331-13 23800397 PMC3697808

[B69] Srinivasan A. Lopez-Ribot J. L. Ramasubramanian A. K. (2015). Microscale microbial culture. *Future Microbiol.* 10 143–146. 10.2217/fmb.14.129 25689525 PMC4690529

[B70] Stewart E. J. (2012). Growing unculturable bacteria. *J. Bacteriol.* 194 4151–4160. 10.1128/jb.00345-12 22661685 PMC3416243

[B71] Tang X. (2018). A new method for the cell culture of anaerobic bacteria: *P. gingivalis*- or P. endodontalis-Mediated Pathways in RAW264.7 Cells. *Biomed. J. Sci. Techn. Res.* 5 2018.

[B72] Usman H. Davidson S.-L. Manimaran N. H. Nguyen J. T. Bah A. Seth R. (2021). Design of a well-defined poly(dimethylsiloxane)-based microbial nanoculture system. *Mater. Today Commun.* 27:102185.

[B73] Versluis D. De J. Bello González T. Zoetendal E. G. Passel M. W. J. V. Smidt H. (2019). High throughput cultivation-based screening on porous aluminum oxide chips allows targeted isolation of antibiotic resistant human gut bacteria. *PLoS One* 14:e0210970. 10.1371/journal.pone.0210970 30653573 PMC6336267

[B74] Villa M. M. Bloom R. J. Silverman J. D. Durand H. K. Jiang S. Wu A. (2019). High-throughput isolation and culture of human gut bacteria with droplet microfluidics. *bioRxiv* [Preprint] bioRxiv 630822, 10.7554/eLife.56998 32553109 PMC7351490

[B75] Wang Z. Volinsky A. A. Gallant N. D. (2014). Crosslinking effect on polydimethylsiloxane elastic modulus measured by custom-built compression instrument. *J. Appl. Polym. Sci.* 134:2014.

[B76] Warren A. D. Kwong G. A. Wood D. K. Lin K. Y. Bhatia S. N. (2014). Point-of-care diagnostics for noncommunicable diseases using synthetic urinary biomarkers and paper microfluidics. *Proc. Natl. Acad. Sci. U.S.A.* 111:3671. 10.1073/pnas.1314651111 24567404 PMC3956200

[B77] Weibel D. B. Diluzio W. R. Whitesides G. M. (2007). Microfabrication meets microbiology. *Nat. Rev. Microbiol.* 5 209–218. 10.1038/nrmicro1616 17304250

[B78] Weibel D. B. Whitesides G. M. (2006). Applications of microfluidics in chemical biology. *Curr. Opin. Chem. Biol.* 10 584–591. 10.1016/j.cbpa.2006.10.016 17056296

[B79] Whitesides G. M. (2006). The origins and the future of microfluidics. *Nature* 442 368–373. 10.1038/nature05058 16871203

[B80] Wondraczek L. Pohnert G. Schacher F. H. Köhler A. Gottschaldt M. Schubert U. S. (2019). Artificial microbial arenas: materials for observing and manipulating microbial consortia. *Adv. Mater.* 31:1900284. 10.1002/adma.201900284 30993782

[B81] Xia Y. Whitesides G. M. (1998). Soft Lithography. *Angew. Chem. Int. Ed.* 37 550–575. 10.1002/(SICI)1521-3773(19980316)37:5<550::AID-ANIE550>3.0.CO;2-G 29711088

[B82] Xu H. S. Roberts N. Singleton F. L. Attwell R. W. Grimes D. J. Colwell R. R. (1982). Survival and viability of nonculturable*Escherichia coli* andVibrio cholerae in the estuarine and marine environment. *Microb. Ecol.* 8 313–323. 10.1007/bf02010671 24226049

[B83] Zengler K. Toledo G. Rappé M. Elkins J. Mathur E. J. Short J. M. (2002). Cultivating the uncultured. *Proc. Natl. Acad. Sci. U.S.A.* 99 15681–15686. 12438682 10.1073/pnas.252630999 PMC137776

[B84] Zhang X. Y. Sun K. Abulimiti A. Xu P. P. Li Z. Y. (2019). Microfluidic System for Observation of Bacterial Culture and Effects on Biofilm Formation at Microscale. *Micromachines (Basel)* 10:606. 10.3390/mi10090606 31547458 PMC6780771

[B85] Zhen X. Lundborg C. S. Sun X. Hu X. Dong H. (2019). Economic burden of antibiotic resistance in ESKAPE organisms: a systematic review. *Antimicrob. Resist. Infect. Control* 8:137. 10.1186/s13756-019-0590-7 31417673 PMC6692939

[B86] Zhou N. Sun Y.-T. Chen D.-W. Du W. Yang H. Liu S.-J. (2019). Harnessing microfluidic streak plate technique to investigate the gut microbiome of *Reticulitermes chinensis*. *MicrobiologyOpen* 8:e00654. 10.1002/mbo3.654 29897677 PMC6436436

[B87] Zhou W. Le J. Chen Y. Cai Y. Hong Z. Chai Y. (2019). Recent advances in microfluidic devices for bacteria and fungus research. *TrAC Trends Anal. Chem.* 112 175–195.

[B88] Zinchenko A. Devenish S. R. A. Kintses B. Colin P.-Y. Fischlechner M. Hollfelder F. (2014). One in a million: flow cytometric sorting of single cell-lysate assays in monodisperse picolitre double emulsion droplets for directed evolution. *Anal. Chem.* 86 2526–2533. 10.1021/ac403585p 24517505 PMC3952496

